# Yin Yang-1 increases apoptosis through Bax activation in pancreatic cancer cells

**DOI:** 10.18632/oncotarget.8654

**Published:** 2016-04-08

**Authors:** Jing-Jing Zhang, Yi Zhu, Chuang Yang, Xian Liu, Yun-Peng Peng, Kui-Rong Jiang, Yi Miao, Ze-Kuan Xu

**Affiliations:** ^1^ Pancreas Institute of Nanjing Medical University, Nanjing 210029, People's Republic of China; ^2^ Pancreas Center, The First Affiliated Hospital of Nanjing Medical University, Nanjing 210029, People's Republic of China; ^3^ Department of General Surgery, The First Affiliated Hospital of Nanjing Medical University, Nanjing 210029, People's Republic of China

**Keywords:** Yin Yang-1, pancreatic cancer, apoptosis, Bax

## Abstract

The transcriptional regulator Yin Yang-1 (YY1) is a tumor suppressor known to be overexpressed in pancreatic cancer. We found that overexpression of YY1 promoted apoptosis and increased the expression and mitochondrial localization of the pro-apoptotic Bax protein in pancreatic cancer cell lines. Luciferase reporter, electrophoretic mobility shift (EMSA), and chromatin immunoprecipitation (ChIP) assays revealed binding of YY1 to the BAX promoter. Moreover, YY1 promoted pancreatic cancer cell apoptosis through Bax transcriptional activation and subsequent translocation of Bax to the mitochondrial membrane, leading to cytochrome c release, and caspase activation.YY1 and BAX are co-expressed in pancreatic cancer tissues and higher BAX expression predicts better outcomes for patients. The ability of YY1 to promote apoptosis in pancreatic cancer cells suggests it may represent a valuable diagnostic and therapeutic target.

## INTRODUCTION

Pancreatic cancer is among most deadly cancers with an extremely short survival time (6 months) and a 3% 5 yearsurvival rate [1]. At the time of diagnosis, more than 80% of patients are already metastatic or have locally advanced cancer, and only 10% to 15% are eligible for surgical resection [2]. Although the drug gemcitabine has been approved in the treatment of pancreatic cancer, its efficacy is weak, and its improvement of overall survival is marginal. Anti-apoptotic Bcl-2 family proteins are often overexpressed in human cancers and are thought to increase resistance to chemotherapeutic drugs [3, 4]. A better understanding of these anti-apoptotic mechanisms could lead to development of potent and nontoxic strategies to overcome them, improving clinical outcomes for pancreatic cancer patients.

The transcriptional regulator Yin Yang-1 (YY1) is a member of the GLI-Kruppel family of zinc finger proteins that are involved in processes such as embryogenesis, cellular proliferation, DNA replication, and differentiation [5–7]. As its name suggests, YY1 can activate or inactivate gene expression depending on its interacting partners and functions as a master of the epigenetic regulatory network [7]. Overexpression of YY1 has been observed in prostate, ovarian, and colon cancer [8–11].YY1 has been proposed as a potential prognostic factor for cancer patients, based on its role in cancer development [8, 11]. Our previous study revealed that YY1 suppresses cell invasion and metastasis in pancreatic cancer by downregulating MMP10 expression [12]. These studies indicate that YY1 functions as a tumor suppressor in pancreatic cancer.

In this study, we overexpressed YY1 in pancreatic cancer cells and found that it promotes apoptosis via upregulating Bax transcription and subsequent activation of Bax by translocation from the cytosol to the mitochondrial membrane.

## RESULTS

### YY1 over-expression strongly induces apoptosis in pancreatic cancer cells *in vitro*

**Figure 1:**
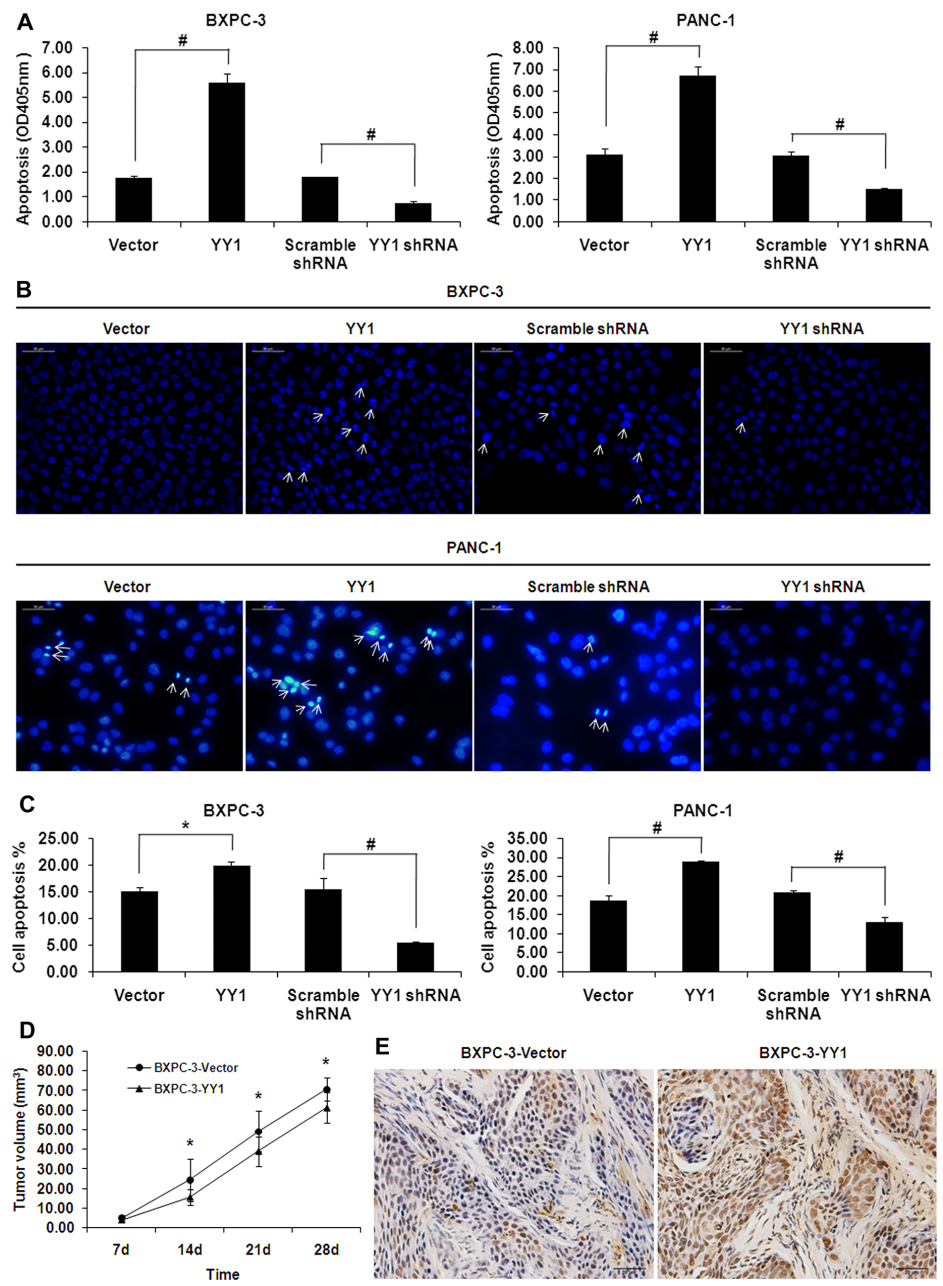
YY1 overexpression induces apoptosis in pancreatic cancer cells *in vitro* and *in vivo* **A.** DNA fragmentation was measured by Cell Death ELISA. Results are representative of three independent experiments and are presented as the mean ± SD (bars). *p< 0.05; #*p*< 0.01. **B.** Changes in nuclear morphology were measured by Hoechst 33258 staining. Arrowheads indicate apoptotic cells characterized by morphological changes such as chromatin condensation and nuclear fragmentation. Results are representative of three independent experiments. **C.** Percentages of apoptotic cells in Annexin V-FITC/PI staining assay were detected by flow cytometry. Results are representative of three independent experiments and are presented as the mean ± SD (bars). *p< 0.05; #*p*< 0.01. **D.** Volumes of tumors grown from BXPC-3-Vector and BXPC-3-YY1 cells bilaterally injected into the flank region of the mice (1.5×10^6^ cells/100 μl per flank). Data are presented as means ± SD of tumors for each group. *p<0.05. **E.** Apoptosis in mouse xenograft tumor tissues was detected by *in situ* TUNEL assay.

We employed DNA fragmentation assays to evaluate the effects of YY1 overexpression on cell apoptosis *in vitro*. YY1 overexpression induced DNA fragmentation in BXPC-3 and PANC-1 cells (Figure 1A). By contrast, YY1 knockdown inhibited the DNA fragmentation. We further confirmed that YY1 overexpression induced apoptosis by detecting and quantifying nuclear morphological changes typical of apoptosis (Hoechst 33258 staining), such as chromatin condensation and nuclear fragmentation, while YY1 knockdown had the opposite effect (Figure 1B). The percentage of apoptotic cells detected by flow cytometry increased in YY1 overexpressing BXPC-3 and PANC-1 cells (Figure 1C). These results indicate that upregulation of YY1 increases apoptotic cell death in pancreatic cancer cells.

### YY1 overexpression strongly induces apoptosis in pancreatic cancer cells *in vivo*

Tumors in mice injected with tumor cells transfected with BXPC-3-Vector were larger than those with BXPC-3-YY1 (Figure 1D). TUNEL staining of mouse tumor tissues indicated the percentage of apoptotic cells was higher in the BXPC-3-YY1 group than in the BXPC-3-Vector control group (Figure 1E, quantitation shown below). The TUNEL assay results were consistent with the DNA fragmentation assay and Hoechst 33258 staining showing that YY1 over-expression induced apoptosis in BXPC-3 pancreatic cancer cells.

### YY1 overexpression promotes apoptosis through mitochondrial cytochrome c release and caspase activation

**Figure 2:**
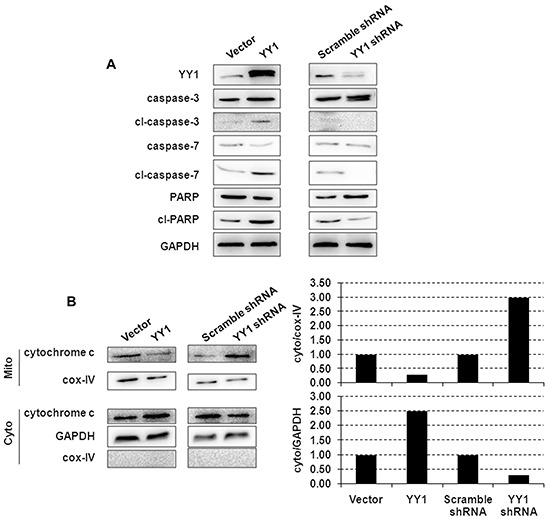
Apoptosis-inducing effect of YY1 overexpression is mediated through mitochondria and caspase **A.** Cleavage of caspases-3 and -7 and PARP in YY1 overexpressing or knockdown BXPC-3 cells was measured by Western blot. Results are representative of three independent experiments. **B.** Cytochrome c localization to mitochondrial membrane and cytosolic fractions was measured by Western blot. Mitochondrial marker Cox-IV (cytochrome oxidase IV) was used as a loading control, and each blot was quantified by densitometry (right panel) to assess the translocation of proteins. Results are representative of three independent experiments.

We next investigated the effect of YY1 on activation of caspases. YY1 overexpression induced cleavage of caspases-3 and -7 and PARP in BXPC-3 cells (Figure 2A), consistent with the DNA fragmentation results (Figure 1A). YY1 over-expression induced cytochrome c release from the mitochondria to cytosol, the key event in apoptosis, as evident by a decrease of mitochondrial cytochrome c and an increase in cytosolic cytochrome c (Figure 2B).

### Bax activation is responsible for YY1-induced apoptosis

**Figure 3:**
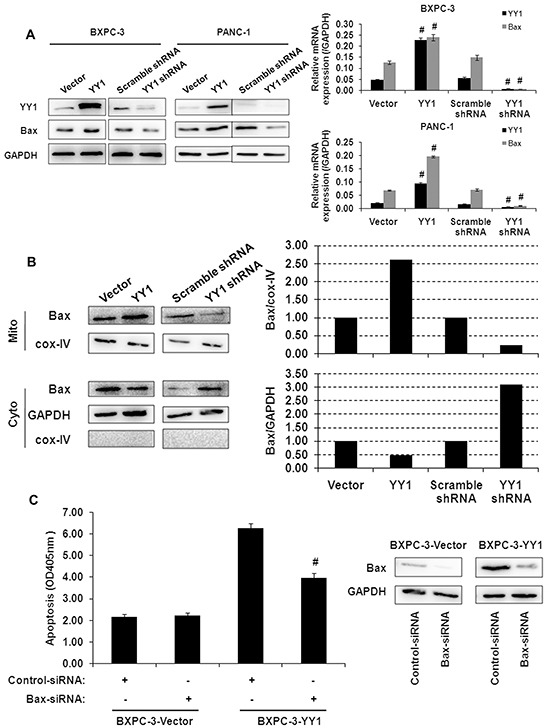
Activation of Bax correlates with apoptosis induced by YY1 overexpression **A.** Bax expression in YY1 overexpressing or knockdown BXPC-3 and PANC-1 cells was measured by quantitative RT-PCR and Western blot. Results are representative of three independent experiments and are presented as the mean ± SD (bars). #*p*< 0.01. **B.** Bax expression in mitochondrial membrane and cytosolic fractions of BXPC-3 cells was measured by Western blot. Each blot was measured by densitometry (right panel) to assess the translocation of proteins. Results are representative of three independent experiments. **C.** YY1 overexpressing BXPC-3 cells were transfected with Bax siRNA for 48 h, after which the extent of apoptosis (left panel) and Bax protein expression levels (right panel) were measured. Results are representative of three independent experiments and are presented as the mean ± SD (bars). #*p*< 0.01.

Activation of the multi-domain molecule Bax (i.e., by conformational change, mitochondrial translocation and oligomerization) leads to cytochrome c release from mitochondria [13]. We found that YY1 overexpression increased, while YY1 knockdown decreased, Bax mRNA and protein expression in whole cell lysates (Figure 3A). Moreover, YY1 overexpression induced Bax translocation from the cytosol to the mitochondria in BXPC-3 cells (Figure 3B). Bax siRNA knockdown partially reversed the induction of apoptosis by YY1 overexpression (Figure 3C). This result indicates that Bax activation is responsible, at least in part, for the induction of apoptosis by the overexpression of YY1.

### Correlation between YY1 and BAX mRNA expression in pancreatic cancer tissues

**Figure 4:**
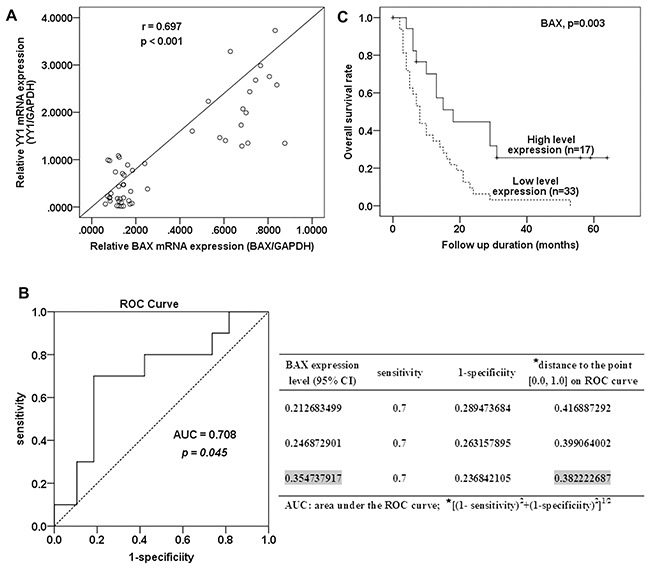
Correlation between BAX expression and patient survival **A.** A scatter diagram for correlation between YY1 and BAX mRNA expression in 50 pancreatic cancer tissues. **B.** ROC curve for BAX expression and cut-off value selection for high and low level BAX expression. **C.** Kaplan-Meier survival curves for 50 patients with pancreatic cancer according to their BAX mRNA expression status. The *p* value was calculated by the Log-rank test.

We measured YY1 and BAX mRNA levels in 50 pancreatic cancer samples using quantitative RT-PCR. YY1 expression levels correlated with BAX expression levels (Figure 4A, r=0.697, *p* <0.001). These results, combined with those of the *in vitro* experiments, suggest that YY1 increases BAX expression, thereby inducing apoptosis in pancreatic cancer.

### Correlation between BAX expression and patient survival

Fifty pancreatic cancer patients were enrolled in the survival analysis. Forty-four patients died, while the remaining 6 were alive at the last follow up (31 March 2013). To determine the BAX expression level cut-off value for survival analysis, the patients were divided into two groups: short-term survivors (survival period < 24 months) and long-term survivors (≥ 24 months). The threshold value of 0.355 was chosen as the cut-off value for high and low BAX expression, because 0.355 (within 95% confidence interval (CI), 0.2-0.4, of BAX mRNA expression) was on the receiver operating characteristic (ROC) curve closest to (0.0, 1.0). This maximized both sensitivity and specificity for the survival outcome (Figure 4B). The area under the ROC curve (AUC) was 0.708 (95% CI, 0.522-0.894, *p*= 0.045).

Kaplan-Meier survival curves shows that patients with high BAX expression (≥0.355) had longer post-operative survival than those with low expression (<0.355) (Figure 4C, *p*= 0.003, log rank test). The two-year survival of patients with high expression of BAX was 41.2%, compared to 9% for patients with low expression. These results indicate that higher expression of BAX predicted better outcome in pancreatic cancer patients.

### YY1 binds to and activates the bax promoter

**Figure 5:**
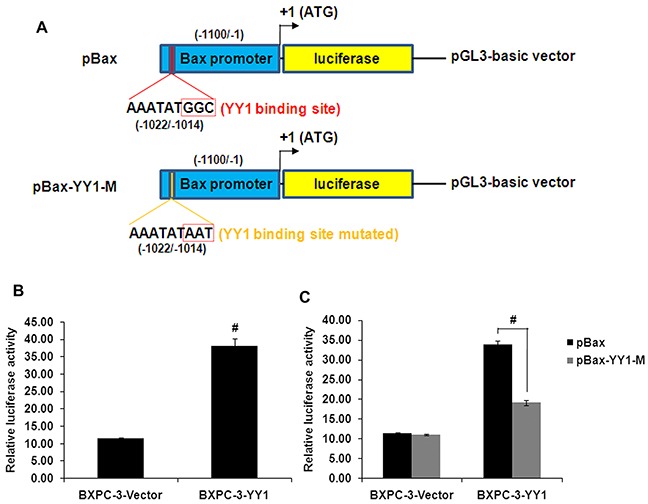
YY1 overexpression increases Bax promoter activity through binding to the presumed YY1 binding site **A.** Schematic diagram of the luciferase reporter construct containing the human *Bax* promoter (pBax) and the mutant construct (pBax-YY1-M) containing the *Bax* promoter in which the presumed YY1 binding site was mutated. **B.** Luciferase activity of *Bax* promoter in YY1 overexpressing BXPC-3 cells was increased compared with control cells. Results are representative of three independent experiments and are presented as mean ± SD (bars). #*p*<0.01. **C.** Luciferase activity of the *Bax* promoter was decreased when the presumed YY1 binding site (nucleotides -1022 to -1014) was mutated. Results are representative of three independent experiments and are presented as the mean ± SD (bars). #*p*< 0.01.

To clarify whether YY1 is involved in *Bax* gene transcription, luciferase activity assays were performed. The luciferase activity was significantly higher in YY1 overexpressing BXPC-3 cells than in BXPC-3-Vector control cells (Figure 5B). Moreover, the luciferase activity of the *Bax* promoter in YY1 overexpressing BXPC-3 cells was decreased when the presumed YY1 binding site (nucleotides -1022 to -1014) was mutated (Figure 5C). These results suggest thatYY1 is a transcriptional activator of the *Bax* gene.

**Figure 6:**
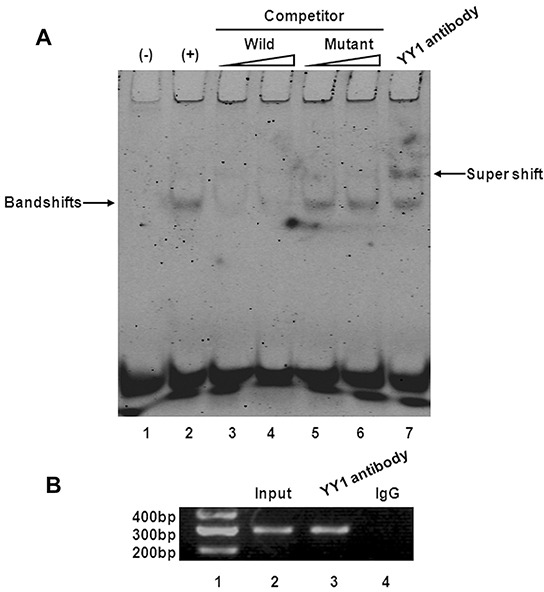
YY1 binds to Bax promoter *in vitro* and *in vivo* **A.** EMSA and supershift assay of YY1 binding to *Bax* promoter. The wild-type probe was incubated without (lane 1) or with (lane 2) BXPC-3-YY1 cell nuclear proteins in the absence or presence of unlabeled probe (lanes 3-6). Lanes 3 and 4 contain the wild-type probe, and lanes 5 and 6 contain the mutant probe, each at 50- and 100-fold molar excess. A supershift assay was performed using an anti-YY1 antibody (lane 7). **B.** ChIP assay of YY1 binding to *Bax* promoter. Lane 1, DNA marker; lane 2, input DNA; lane 3, DNA from BXPC-3-YY1 cells immunoprecipitated with anti-YY1 antibody; lane 4, DNA from BXPC-3-YY1 cells immunoprecipitated with normal rabbit IgG.

To confirm that YY1 binds to the *Bax* promoter in BXPC-3 cells, we synthesized and labeled an oligonucleotide spanning the -1022 to -1014 region with an additional seven nucleotides on each side (i.e., from -1031 to -1006) and used it as a probe in EMSA experiments. As shown in Figure 6A (lane 2), a slower-migrating complex appeared when YY1 overexpressing BXPC-3 cell nuclear extracts were incubated with the digoxigenin-11-ddUTP-labeled wild-type probe (−1031 to -1006 of the *Bax* promoter). The complex was inhibited by a molar excess of unlabeled wild-type competitor (Figure 6A, lanes 3, 4). In contrast, the mutant competitor (mutation of -1022 to -1014) reduced the inhibitory effect (Figure 6A, lanes 5, 6). In supershift analyses, the DNA-protein complex could be supershifted by addition of YY1 antibody (Figure 6A, lane 7). These results suggest YY1 specifically binds to the *Bax* promoter *in vitro*.

Chromatin from YY1 overexpressing BXPC-3 cells was immunoprecipitated with anti-YY1 antibody or normal rabbit IgG, and the purified DNA was then analyzed by PCR using primers specific for the region containing the YY1-binding site. The presence of the promoter-specific DNA region before immunoprecipitation was confirmed by PCR (input). PCR product was observed in YY1 immunoprecipitation groups (Figure 6B), consistent with EMSA results indicating the presence of an “*in vivo*” interaction between YY1 and the *Bax* promoter.

## DISCUSSION

In this study, we demonstrated that YY1overexpression in pancreatic cancer cells increases apoptosis. Bax activates both intrinsic and extrinsic apoptotic pathways [14–16]. Bax translocation from the cytosol to the mitochondria induces apoptotic cell death via the intrinsic apoptotic pathway [15, 17, 18]. Bax activation involves conformational change, insertion into the mitochondrial outer membrane, homo- or hetero-oligomerization, induction of mitochondrial outer membrane permeability (MOMP), and release of cytochrome c from the inter-membrane space [15, 19–21]. Moreover, Bax protein levels are critical for promoting its translocation to the mitochondria [15]. In this study, we found that YY1 overexpression increased Bax mRNA and protein expression and thus induced Bax translocation from the cytosol to the mitochondria in pancreatic cancer cells. In addition, YY1 expression correlated with BAX expression in pancreatic cancer tissues, also suggesting that YY1 positively regulates the expression of BAX. Bax knockdown was found to partially reverse the induction of apoptosis by YY1 overexpression, indicating that Bax activation may be responsible, at least in part, for the induction of apoptosis by YY1.

YY1 appears to directly activate *Bax* gene transcription. Our luciferase activity assays showed that overexpression of YY1 increased the activity of the *Bax* promoter. Our detailed EMSA and ChIP analyses demonstrate that YY1 binds directly to the presumed YY1 binding site *in vitro* and *in vivo*. These results indicate that YY1 binds to the *Bax* promoter to enhance gene transcription.

In summary, we found that YY1 overexpression increases apoptosis in pancreatic cancer cells through upregulation of *Bax* transcription and subsequent translocation of Bax from the cytosol to the mitochondrial membrane, where it is activated. Although YY1 also exert other effects, the results of the present study indicate that YY1 promotes apoptosis in pancreatic cancer cells and may represent a valuable diagnostic and therapeutic target.

## MATERIALS AND METHODS

### Cell lines and cell culture

The stable YY1 overexpressing BXPC-3 cell line (BXPC-3-YY1), YY1 knockdown BXPC-3 cell line (BXPC-3-YY1 shRNA), and their control cell lines (BXPC-3-Vector, BXPC-3- Scramble shRNA) were prepared previously [12]. The cells were grown in Dulbecco's modified Eagle's medium (DMEM) (Wisent Inc., Montreal, Canada) supplemented with 10% fetal calf serum (FBS) (Wisent), 10 mM HEPES (Wisent), 2 mM L-glutamine (Wisent), 1 mM pyruvate sodium (Wisent), 100 units/ml penicillin (Wisent), and 100 μg/ml streptomycin (Wisent) at 37°C in a humidified atmosphere containing 95% air and 5% CO_2_.

### Preparation of YY1 overexpression/YY1 knockdown PANC-1 cells

YY1-overexpression lentiviruses, YY1-knockdown lentiviruses, and their control lentiviruses were prepared previously [12]. Stable cell lines (PANC-1-Vector, PANC-1-YY1, PANC-1-Scramble shRNA and PANC-1-YY1 shRNA) were selected by culturing in media containing 5 μg/ml puromycin (Sigma, St Louis, MO, USA). YY1 expression was confirmed by qRT-PCR and Western blot.

### DNA fragmentation assay

DNA fragmentation was analyzed using the Cell Death Detection ELISA^Plus^ kit (Roche Diagnostics GmbH, Mannheim, Germany) according to the manufacturer's instructions. Briefly, cells (10^4^ cells/well in 200 μl) were incubated in serum-free media for 12 hat 37°C. Before and after lysis, cells were centrifuged, and a 20 μl aliquot of the supernatant was analyzed. The extent of apoptosis was determined by absorbance at 405 nm.

In the Bax blockage experiments, YY1 over-expressing BXPC-3 cells were transfected with Bax or negative control siRNA (ThermoFisher Scientific, Rockford, IL, USA, final concentration 30 nM) using Lipofectamine 2000 (ThermoFisher Scientific), according to the manufacturer's protocol. At 24 h after transfection, DNA fragmentation assays were performed as described above.

### Hoechst 33258 staining

To assess changes in nuclear morphology during apoptosis, staining using Hoechst 33258 (Beyotime, Shanghai, China) was performed. Cells were seeded into a 6-well cell culture plate with sterile coverslips and incubated overnight at 37°C. After culturing in serum-free media for 24 h, cells on the coverslips were fixed with 4% paraformaldehyde in PBS for 30 min at room temperature and then stained with Hoechst 33258 for 5 min. Coverslips were mounted with Antifade Mounting Medium (Beyotime) and observed under a fluorescence microscope (Nikon, Eclipse 80i,Tokyo, Japan). Apoptotic nuclei were identified by morphologic changes such as chromatin condensation and nuclear fragmentation.

### Annexin V-fluorescein isothiocyanate (FITC)/propidium iodide (PI) staining assay

After culture in serum-free media for 24 h, cells were harvested and washed with cold PBS. Levels of phosphatidylserine on the cell surface were estimated by using the Annexin V-FITC and PI apoptosis detection kit according to the manufacturer's instructions (Beyotime). Apoptotic cells were analyzed in a flow cytometer (Beckman Coulter, Brea, CA, USA) using FL1 (excitation 488 nm, green) and FL3 (excitation 585 nm, red) channels. A minimum of 10,000 events were collected for each sample. Results are expressed as the percentage of apoptotic cells relative to total cells.

### BXPC-3 xenograft tumor model

All animal research procedures conformed to the guidelines for The Care and Use of Laboratory Animals published by the National Institutes of Health and were approved by The Laboratory Animals Care and Use Committee of Nanjing Medical University. Four-week-old female nude mice (BALB/cA-nu) were purchased from the Shanghai Experimental Animal Center (Chinese Academy of Sciences, Shanghai, China). Eighteen mice were randomly divided into two groups. For the xenograft subcutaneous implant tumor model, BXPC-3-Vector and BXPC-3-YY1 cells were bilaterally injected subcutaneously into the flank region (1.5 × 10^6^ cells/100 μl per flank). Bidimensional tumor measurements were taken with calipers once weekly. Tumor volume was calculated using the formula (width^2^×length)/2. Four weeks later, mice were euthanized and subcutaneous tumors were removed, fixed in 4% paraformaldehyde, and embedded in paraffin after 24 h for the *in situ* terminal deoxynucleotidyl transferase dUTP nick end labeling (TUNEL) assay.

### TUNEL assay

To investigate cell apoptosis induced by YY1 over-expression *in vivo*, an *in situ* TUNEL assay (Beyotime) was performed according to the manufacturer's instructions. Briefly, after deparaffinization and rehydration, tissue sections were permeabilized by proteinase K at room temperature for 15 min. Slides were washed three times in PBS and then immersed in 3% H_2_O_2_/PBS at room temperature for 20 min to quench endogenous peroxidase activity. After completion of TdT-end labeling, tissue sections were placed in stop buffer for 10 min at room temperature to terminate the TdT enzyme reactions and washed three times in PBS. Sections were sequentially incubated with antibody solution and conjugate. Finally, slides were developed in 3, 3′ diaminobenzidine (DAB) chromogen and counterstained with hematoxylin. The mean of TUNEL-positive cells was calculated by counting cells from four random fields for each sample.

### Quantitative RT-PCR (qRT-PCR)

Total RNA samples were prepared using PureZOL RNA Isolation reagent (Bio-Rad, Hercules, CA, USA) according to the manufacturer's instructions. After spectrophotometric quantification, 1 μg of total RNA was used for reverse transcription (RT) in a final volume of 20 μl with iScript cDNA Synthesis Kit (Bio-Rad) according to the manufacturer's instructions. Quantitative PCR was performed using TaqMan Gene Expression Assay (ThermoFisher Scientific, Rockford, IL, USA) in a StepOne Plus Real-time PCR System (ThermoFisher Scientific). Reactions were performed in a volume of 10 μl containing 1 μl diluted cDNA, 20× TaqMan Gene Expression Assay Mix and 2× TaqMan Universal PCR Master Mix. Thermal cycling conditions consisted of an initial denaturation step at 95°C for 10 min, 40 cycles at 95°C for 15 s, and 60°C for 1 min. TaqMan Gene Expression Assay Mixes were used to detect *YY1* and *Bax* (product numbers Hs00231533_m1 and Hs00180269_m1, respectively), while human *GAPDH*(product number Hs02758991_g1) was measured to calibrate the original mRNA concentration. Relative gene expression was calculated by subtracting the Ct value of *YY1* or *Bax* (target) and *GAPDH* (control) gene by the 2^−ΔΔCT^ method [22]. Each quantitative PCR was performed in triplicate and independently repeated three times.

### Western blotting

Total cell lysates were collected as described previously [23]. Protein concentrations were measured using the DC protein assay kit (Bio-Rad). Equal amounts of protein were electrophoresed on 12% SDS-PAGE and transferred to a PVDF membrane (Bio-Rad). Nonspecific protein interactions were blocked by incubation in 5% nonfat dry milk in 0.1% Tween 20 (TBST) buffer at room temperature for 1 h and then membranes were washed with TBST. Membranes were incubated at 4°C overnight with primary antibodies in fresh blocking buffer. Anti-GAPDH was purchased from Santa Cruz Biotechnology (Dallas, Texas, USA) and anti-YY1 from Abcam (Cambridge, MA, USA). The antibodies to caspase-3, cleaved caspase-3, caspase-7, cleaved caspase-7, PARP, cleaved-PARP, cytochrome c, Bax and cox-IV were purchased from Cell Signaling Technology (Danvers, MA, USA). The blots were then washed and incubated with HRP-conjugated secondary antibodies (Beyotime) for 1 h at room temperature. Bands were visualized with Immobilon Western Chemilum HRP substrate (Merck Millipore, Darmstadt, Germany) using the Fluorchem E System (ProteinSimple, Santa Clara, CA, USA). Prestained markers (ThermoFisher Scientific) were used as internal molecular weight standards. Each blot was independently repeated three times.

### Subcellular fractionation

For preparation of cell mitochondrial and cytosolic fractions, we used a mitochondria extraction kit (ThermoFisher Scientific) according to the manufacturer's instructions. Briefly, cells were resuspended in lysis buffer and then disrupted with a Dounce homogenizer. Homogenates were centrifuged at 700 × *g* for 10 min at 4°C to pellet nuclei and cell debris. Supernatants were subsequently centrifuged at 12,000 × *g* for 15 min at 4°C, and the cytosolic fractions (supernatants) were collected. Pellets (heavy membranes enriched with mitochondria) were boiled with SDS-PAGE sample buffer (Beyotime) and analyzed by Western blotting. To determine the quality of cytosolic and mitochondrial separation, both fractions were assessed by immunoblotting for the mitochondrial marker cox-IV.

### Patients and pancreatic tissues

Pancreatic tissue samples were obtained from fifty patients who underwent pancreaticoduodenectomy for pancreatic cancer at the first Affiliated Hospital of Nanjing Medical University, China, between 2006 and 2012. No chemotherapy or radiation therapy was administered before tumor excision. Written informed consent was obtained from all patients undergoing surgery and the Ethics Committees of the first Affiliated Hospital of Nanjing Medical University approved the study. Tissue samples were collected from these patients during surgery. Immediately (within 5 min) upon surgical removal, each tissue sample was cut in two; one was snap-frozen in liquid nitrogen until use and the other was fixed in 5% formalin and embedded in paraffin after 24 hr. A pathologist examined all tissue samples histologically to confirm the diagnosis. The fifty patients were followed up regularly until 31 May 2013. Patients’ overall survival was defined as the time between surgery and death or the last follow-up date. None of the 50 selected patients died within one month after surgery.

### Construction of reporter gene plasmids

A luciferase reporter construct containing the human *Bax* promoter (−1100/−1, upstream of translation initiation sites, TIS) was prepared using the pGL3-basic vector (Promega, Madison, WI, USA). A DNA fragment of the *Bax* promoter region (including restriction enzyme sites) synthesized by GenScript Biotechnology Co., Ltd (Nanjing, China) was subcloned into the *Kpn*I and *Xho*I sites of the pGL3-basic vector to construct the pGL3-Bax-promoter (pBax) recombinant plasmid, which was confirmed by sequencing. The mutant construct pBax-YY1-M, containing the *Bax* promoter in which the presumed YY1 binding site (nucleotides -1022 to -1014) was mutated from AAATATGGC to AAATATAAT, was also constructed.

### Cell transient transfection and luciferase assay

Transfections were performed using Lipofectamine 2000 according to the manufacturer's protocol. Cells were seeded in 12-well cell culture plates (2 × 10^5^/well) 1 day before transfection. Each transfection was performed using 1 μg of the luciferase reporter construct pBax or pBax-YY1-M plus 2.5 ng of the Renilla luciferase reporter vector pRL-SV40 as an internal control (Promega, Madison, WI, USA). 48 h after transfection, cells were washed with PBS and lysed using 1× passive lysis buffer. Firefly and Renilla luciferase activities were measured with a GloMax-20/20 luminometer (Promega) using the Dual-Luciferase Reporter Assay System (Promega). Firefly luciferase activity was normalized to the Renilla luciferase activity. Each experiment was performed in triplicate and independently repeated three times.

### Nuclear protein extraction and electrophoretic mobility shift assay (EMSA)

Nuclear extracts were isolated from YY1 over-expressing BXPC-3 cells with NE-PER Nuclear and Cytoplasmic Extraction Reagents (ThermoFisher Scientific) according to the manufacturer's instructions. Protein concentrations were determined with the DC Protein Assay kit (Bio-Rad). Nuclear extracts were stored at -80°C until use. EMSA was performed using the DIG Gel Shift Kit (Roche) according to the manufacturer's protocol. The sense probe sequences for EMSA were as follows: wild-type probe, 5′-TTCAGATAAAAATATGGCATATTTGGG-3′, corresponding to nucleotides -1031 to -1006 of the *Bax* promoter; mutant probe, 5′-TTCAGATAAAAATATAATATATTTGGG-3′. Double-stranded (ds) probes were synthesized, and the 3′-end of the wild-type probe was labeled with digoxigenin-11-ddUTP. Nuclear extracts (5 μg protein) were incubated with 1 μg poly [d (I-C)], the binding buffer included in the kit and DIG-labeled wild-type probe in the presence or absence of unlabeled probe for 15 min at room temperature. Bound DNA complexes were separated by 5% nondenaturing polyacrylamide gel electrophoresis and transferred to a nylon membrane (Roche). The nylon membranes were cross-linked, and chemiluminescent detection was performed using CSPD. Signals were recorded using the Fluorchem E System.

In supershift analyses, the YY1 antibody (4μg; Abcam) was added to nuclear extracts in gel shift buffer (above) for 1 h at 4°C, followed by addition of the probe. The subsequent protocol steps were the same as above.

### Chromatin immunoprecipitation (ChIP)

ChIP analysis was performed using the EZ ChIP kit (Merck Millipore) according to the manufacturer's instructions with some modifications. To crosslink proteins to DNA, formaldehyde (final concentration 1%) was added to the culture medium of YY1 over-expression BXPC-3 cells and incubated for 10 min at room temperature. Thereafter, a final concentration of 0.125 M glycine was added to stop fixation, and cells were scraped and collected by centrifugation at 700 × *g* for 5 min at 4°C. Cell pellets were treated with Lysis Buffer containing 1× Protease Inhibitor Cocktail II. Aliquots of cell lysates were sonicated to shear DNA into 0.2“1.0-kb fragments, and cellular debris was removed by centrifugation at 14,000 × *g* for 10 min at 4°C. The resultant chromatin-containing solutions were aliquoted (100 μl) and stored at -80°C until use. Chromatin aliquots were precleared with 60 μl of 50% protein G agarose suspension. Samples were then incubated with 10 μg anti-YY1 antibody (Abcam) or normal rabbit IgG (as a control) overnight at 4°C with rotation. Immunocomplexes were mixed with 60 μl of 50% protein G agarose suspension, followed by incubation for 1 h at 4°C with rotation. Beads were collected by brief centrifugation, and the immunocomplexes were eluted by freshly prepared elution buffer (100 mM NaHCO_3_, 1% SDS). Chromatin was then de-crosslinked for 5 h at 65°C. After treatment with RNase A and proteinase K, DNA was purified with spin columns and eluted in 50 μl of elution buffer C. An aliquot (2 μl) of each sample was subjected to PCR analysis using Hot-Start Taq DNA polymerase (Takara, Dalian, China) (32 cycles). The primers used were as follows: sense TTGAGACCAGCCTGACCAAC, antisense GTGCCCCAAATATGCCATAT (product length 297 bp).

### Statistical analysis

All data were representative of at least three independent experiments. Statistical analysis was performed using the SPSS software (Version 15.0). Quantitative data are presented as mean ± SD. Differences in the mean of two samples were analyzed by Student's *t* test. Correlations between YY1 mRNA levels and BAX mRNA levels were analyzed by the Spearman rank correlation test. Receiver operating characteristic (ROC) curve analysis determined the YY1 expression level cut-off value for survival analysis [24–26]. Survival distributions and overall survival rates were determined using the Kaplan-Meier method, and the significance of differences between survival rates was calculated by the Log-rank test. All statistical tests were two-tailed exact tests with a *p* < 0.05 considered significant.
